# A Friction-Inertial-Based Rotary Motor: Design, Modelling and Experiments

**DOI:** 10.3390/ma11060918

**Published:** 2018-05-29

**Authors:** Bo Zhang, Fangxin Chen, Haiyang Li, Zhijiang Du, Lining Sun, Wei Dong

**Affiliations:** State Key Laboratory of Robotics and System, Harbin Institute of Technology, 2 Yikuang Street, Harbin 150080, China; dongway@gmail.com (B.Z.); chenfx@hit.edu.cn (F.C.); lihaiyang_1991@126.com (H.L.); duzj01@hit.edu.cn (Z.D.); lnsun@hit.edu.cn (L.S.)

**Keywords:** friction–inertial driving, load capacity, step loss

## Abstract

A friction–inertial-based rotary motor driven by shear piezoelectric actuators (SPAs) is proposed in this paper, which possesses many superior features, including high resolution, compact size, large load-capacity, and low cost. In order to eliminate the step loss and increase the step size when an external load is applied, the power-function-shape driving signal was used to actuate the rotary motor. According to the step characteristics under this driving signal, two motion modes were observed and defined, namely the stick-shoot motion mode and the stick-slip-shoot motion mode. The former motion mode can realize a large step size while the later one cannot due to the slipping during the rising phase. After analyzing the results from the numerical simulation and the experiment study, it was found that the motion performance of the motor is closely related to the preload and the base number of the driving signal rather than the size of SPAs, which means the motor can be further downsized according to its actual requirements.

## 1. Introduction

Researches on precision engineering and nanotechnology have had many outcomes in recent years which have been widely used in nano-manipulation and nano-positioning, such as scanning probe microscopy and alignment of optics [1,2]. The nanotechnologies employed in precision engineering include compliant mechanisms [3], inchworm actuators [4], and piezoelectric stick–slip actuators, etc. Compared with other actuators, the piezoelectric stick–slip actuator has unique advantages because it is simple to operate and solves the contradiction between high accuracy and large displacement [5,6,7,8].

Rotary motors can be divided into three categories according to the working principle of the piezo actuators, which are rotary motors based on fiber torsional piezo actuators [9], rotary motors based on walking piezo actuators [10], and rotary motors based on shear piezoelectric actuators (SPAs). Compared with others, the rotary motor with SPAs is superior in terms of its simple structure and compact size [11]. As a result, much attention has been paid to SPA-based stick–slip actuators. Van [12] developed a piezo-rotor based on inertial sliding with nano-radial resolution and unlimited range. Howald [13] proposed a piezoelectric inertial stepping motor for multiple rotations, whose maximum speed is 17 μrad/s with the driving signal of 100 V and 1 kHz. Based on the optimized design in [13], Neuman [5] proposed an optimization method of the driving signal to achieve the fastest and most reliable actuator operation. Lambert [8] designed a simple and cheap system to produce a 2 cm travel with a 20 nm resolution, where the relations between inertia and step loss were studied via experiments. In summary, the majority of previous research focused on the resolution and the velocity, but few of them dealt with the load capacity and step characteristics of a rotary motor with an external load. However, load capacity is a challenging issue in practice [14,15,16]. In addition, the miniaturization of rotary motors is also a unique feature in contrast to electromagnetic motors, which means they can be applied in a small space.

The objective of this paper is to conduct a systematic investigation of the motion performance of the stick–slip based rotary motor with SPAs when an external load is applied and to improve the step characteristics without changing the mechanical design (i.e., maintain its compact size). To achieve this goal, we proposed a friction–inertial based rotary motor driven by SPAs and tried to use different driving signals to eliminate the step loss and increase the step size when an external load was applied. It was found that the power-function-shape driving signal is more suitable for the motor. Moreover, the motion performance of the motor is closely related to the preload and the base number of the driving signal rather than the size of the actuation units.

The remainder of this paper is organized as follows. The design of the friction–inertial based rotary motor driven by SPA and the motion modeling are described in Section 2. Also in this section, the conventional driving signal, the saw-tooth wave, was verified unsuitable for the motor when external loads were applied due to the step loss phenomenon. In Section 3, the power-function-shaped driving signal is utilized for the motor to generate a fast and reliable actuating operation. The motion model based on this driving signal with external loads is analyzed by the established model. In Section 4, the results of the numerical simulation and the experiment are presented to verify the feasibility of the proposed method. Finally, some concluding remarks are given in Section 5.

## 2. Experimental Setup and Motion Modeling

### 2.1. Experimental Prototype of the Rotary Motor

**Figure 1:**
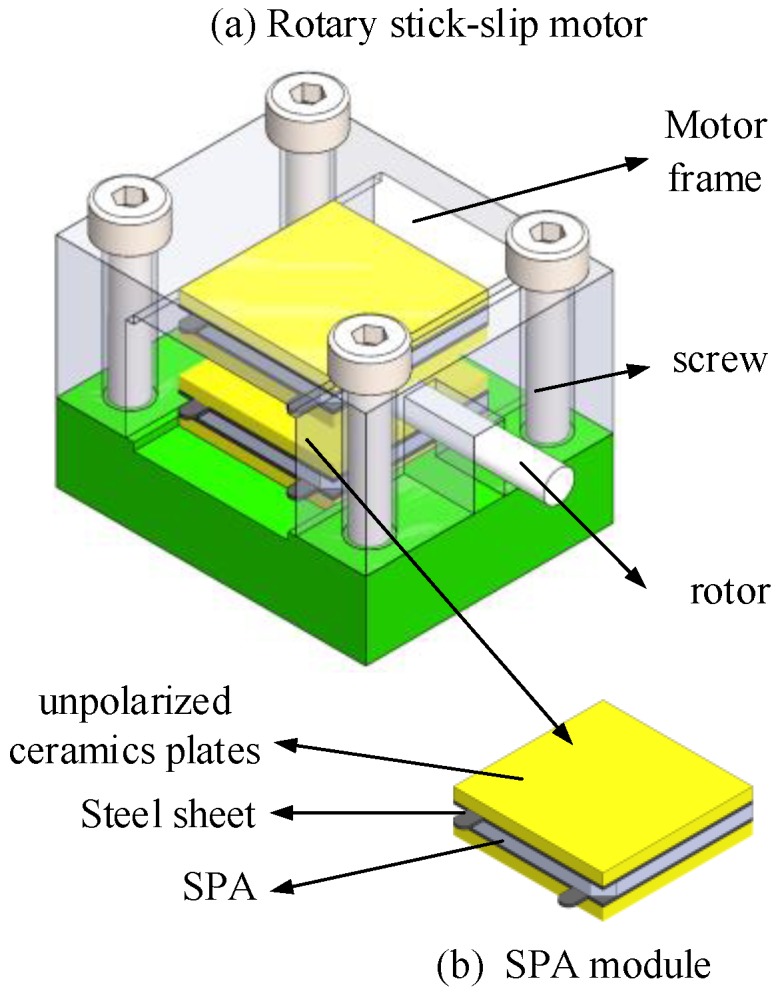
(**a**) Schematic view of the rotary stick–slip motor; (**b**) The SPA module.

The experimental prototype of the rotary motor consists of two SPA modules, a rotor, and a motor frame, as shown in Figure 1. In order to apply a driving voltage on the SPA, each SPA is fixed with two steel sheets by copper conductive adhesives. In addition, two unpolarized ceramic plates are glued outside the steel sheets by epoxy resin to ensure effective insulation and perfect interface between the SPA module and the rotor. The SPA modules and the rotor are mounted in the motor frame which is fixed by four screws. The preload which has a significant influence on the motion performance of the motor is also produced by the four screws.

**Figure 2:**
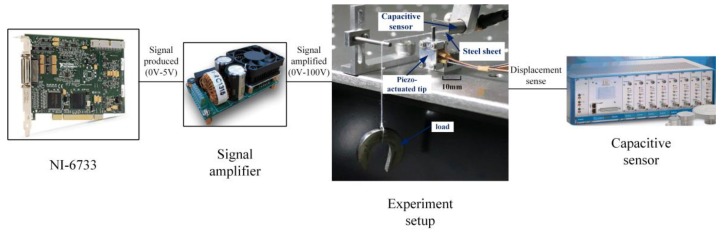
The experimental setup.

The external torsional load is generated by the weight fixed on the rotor, as shown in Figure 2. The steel sheet inserted into the rotor gap is used as the sensing surface of the measurement system, where a capacitive sensor, a product from Micro-Epsilon Company, is utilized to measure the displacement of the rotor. The driving signal is produced by a D/A card (NI-6733 PCI), where various driving signals can be generated and tuned through a LabVIEW program. The output of the driving signal is amplified by the amplifier MX200 (Piezodrive, Australia) before being applied on the motor. 

### 2.2. Motion Modeling

**Figure 3:**
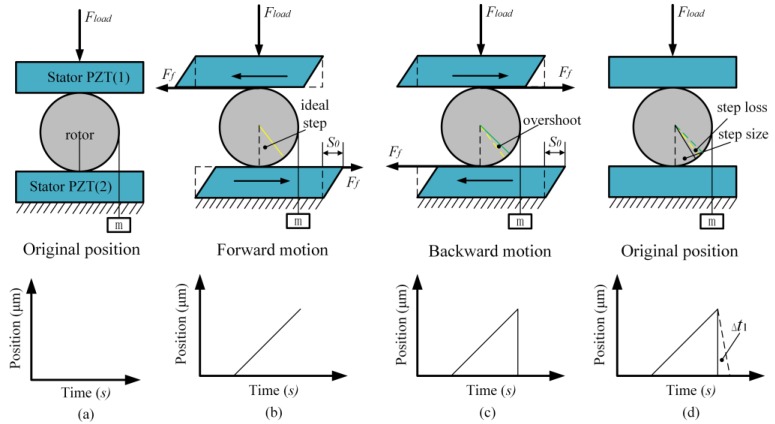
The schematic of the motor motion mechanism with external loads. (**a**) Initial position; (**b**) Sticking phase; (**c**) Slipping phase; (**d**) Overshoot.

The piezo-actuated motor is driven by the friction between the SPA and the rotor. During each motion step, two phases are involved: the sticking phase and the slipping phase. The schematic of the motor’s motion mechanism with an external load is shown in Figure 3. In the sticking phase, the rotor sticks to the contact surface of the SPA (i.e., there is no relative slip), which moves along slowly with the SPA (here we call it in the forward direction). At the end of this phase, the SPA stops immediately while the rotor keeps moving by a small distance *a* due to the inertial effect, which is termed as overshoot. Then, in the slipping phase, the SPAs return to the original position within the time interval Δ*t*_1_, during which the rotor moves in the backward direction by a distance *b*, which is termed as step loss, as shown in Figure 3. Therefore, the actual step size can be calculated as $\varphi =\varphi_{0}+a-b$, where *ϕ*_0_ is the ideal step size during the sticking phase. The overshoot is determined by the instantaneous velocity at the end of the sticking phase and the step loss is determined by the time Δ*t*_1_ and the acceleration of the rotor during this time [5,15]. Note that Δ*t*_1_ is the response time of the SPA, which can be measured through experiments. Distances *a* and *b* are called backlash, which are usually ignored in practice. However, when an external load is applied on the rotor, the step loss is aggravated so as to significantly deteriorate the step response of the motor. Therefore, a motion model which takes backlash into account has to be built.

In the sticking phase, the tangential acceleration of the contact point on the rotor *a*_R_ equals the acceleration of that on the SPA *a*_S_, so we can write,

(1) $$\varepsilon =\frac{a_{S}}{R}=\frac{a_{R}}{R}=\frac{M(t)-M_{load}}{I}$$

with

(2) $$M_{load}=F_{load}\cdot R$$

where *F_load_* is the external load. *R* and *I* are the rotor’s radius and inertia momentum. The acceleration of the SPA is determined by the driving voltage applied on the SPA. In this phase, the acceleration *ε* must meet the following condition,

(3) $$\varepsilon \leq \varepsilon_{c}$$

with

(4) $$\varepsilon_{c}=\frac{M_{\max}-M_{load}}{I}$$

(5) $$M_{\max}=F_{pre}\cdot f_{s}\cdot R$$

where *F_pre_* and *f_s_* are the preload and the static friction coefficient, and *ε_c_* is the critical acceleration, preventing the rotor from slipping.

As we can see from Equation (5), the external load decreases the critical acceleration, in other words, the allowed acceleration in the sticking phase decreases with the external load increasing.

In the slipping phase, the SPAs move back to the original position extremely quickly, during which the rotor gets an opposite torsional force generated by the dynamic friction between the SPA and the rotor. The acceleration can be calculated as below,

(6) $$\varepsilon_{b}=-\frac{M_{b}(t)+M_{load}}{I}$$

with

(7) $$M_{b}(t)=F_{pre}\cdot f_{d}\cdot R$$

where *f_d_* is the dynamic friction coefficient. The minus sign in Equation (6) indicates that the direction of the acceleration is opposite to the acceleration in the sticking phase. The external load increases the acceleration in this phase, which means the step loss is aggravated by the external load applied.

When the motor is actuated by a saw-tooth wave, the instantaneous velocity at the stick phase is small, so the overshoot can be ignored. According to Equation (1), the actual step size of the stick–slip motion with an external load can be calculated as below,

(8) $$\varphi =\varphi_{0}-b$$

with

(9) $$b=\frac{1}{2}\cdot \varepsilon_{b}\cdot t_{b}^{2}.$$

### 2.3. Step Loss in Experiments

**Figure 4:**
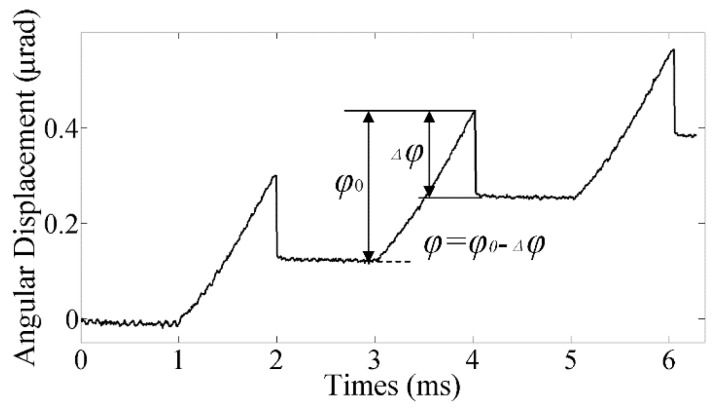
The backlash phenomenon.

**Figure 5:**
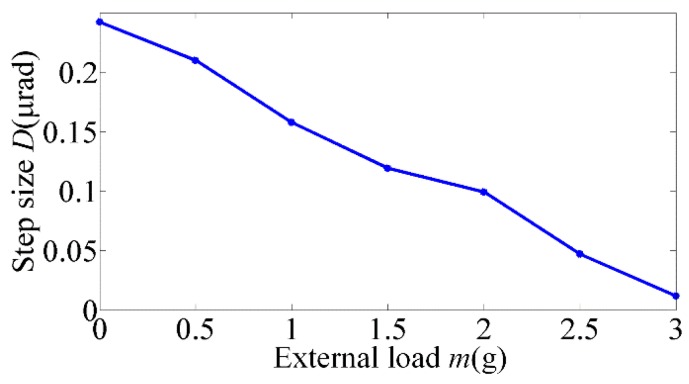
Relation of the external load and step size.

The saw-tooth wave was applied in the experiments to validate the motion model established above and prove the conclusion that the step size will decrease when an external load is applied due to the aggravated step loss. The step characteristics of the rotor are shown in Figure 4, from which we can see that the step loss is extremely noteworthy. The actual step size significantly decreases in contrast to the nominal stroke of the SPA, which will further reduce with the external load increasing, as shown in Figure 5.

As suggested by the results collected from the experiments, the saw-tooth wave is unsuitable for the rotary motor with external loads. In order to improve the motion performance of the rotary motor without changing its mechanical design, the form of the driving signal should be reconsidered.

## 3. Motion Model with Power-Function-Shaped Driving Signals

According to the mechanism of backlash, overshoot and step loss occur simultaneously at the end of the sticking phase. If the time of overshoot is longer than the time of step loss, the step loss will not occur. To satisfy this condition, a higher instantaneous velocity of the rotor at the end of the sticking phase is needed. According to reference [5], the power-function-shaped driving signal can realize an extremely high velocity at its ending stage in contrast to other driving curves. Therefore, the exponential curve is intuitively considered to be the best candidate curve for the rotary motor to achieve a large step size and eliminate step loss. To verify the feasibility of the proposed solution, the analytical model of the motor motion with the power-function-shaped driving signal is established in this section.

**Figure 6:**
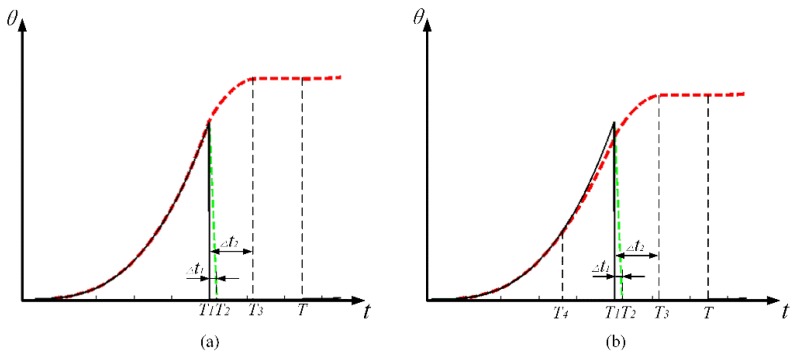
The driving pulse (solid line) and the rotor response (the red dotted line) with specific time sections in two motion models: (**a**) stick–shoot model; (**b**) stick–slip-shoot model.

Different from the saw-tooth wave, the acceleration realized by the exponential curve keeps increasing. Therefore, two distinct motion modes should be discussed respectively according to the condition that whether the maximum acceleration exceeds the critical acceleration. If the maximum acceleration at the end of the sticking phase is smaller than the critical acceleration calculated by Equation (5), the rotor will keep sticking to the SPA. According to the motion state, the repetition period *T* can be divided into three sections, i.e., the rising section, the shooting section, and the pausing section, as shown in Figure 6a, where *T*_1_ represents the rising time, while *T*_2_ and *T*_3_ represent the right margin of the time interval of step loss Δ*t*_1_ and overshoot Δ*t*_2_ respectively. This motion mode is termed as stick–shoot mode in this paper. On the contrary, when the intermediate acceleration in the rising phase (at *T*_4_) is larger than the critical acceleration, as shown in Figure 6b, the rotor will start to slip at *T*_4_. Compared with the first motion mode, the rising phase should be further divided into the sticking section and the slipping section by *T*_4_. This motion mode is termed as stick–slip–shoot mode.

As to the stick–shoot motion mode, the analytical description of the rotor’s angular displacement in the three distinct time intervals is given by the following [5],

(10) $$\begin{array}{ll} \theta (0<t<T_{1})=\frac{S(t)}{R}=\frac{S_{0}}{R}\cdot {\left(\frac{t}{T_{1}}\right)}^{n}, & \left(\mathrm{rising}\;\mathrm{section}\right)\\ \theta (T_{1}<t<T_{3})=\theta (T_{1})+\frac{\omega {\left(T_{1}\right)}^{2}}{2\varepsilon_{b}}, & \left(\mathrm{shooting}\;\mathrm{section}\right)\\ \theta (T_{3}<t<T)=\theta (T_{3}), & \left(\mathrm{pausing}\;\mathrm{section}\right) \end{array}$$

where *S*_0_ is the free stroke of the SPA with the applied voltage. The base number *n* is larger than zero. It can be seen that the first formula in Equation (10) is obtained according to the power-function-shape curve (There is no slipping during the rising section, thus the displacement of the rotor is equal to the deformation of the SPA, i.e., is proportional to the amplitude of the driving signal). As for the second one, during the shooting section, the rotor receives the load and friction force in the backward direction, thus the deceleration of the rotor is constant and the motion of the rotor can be described applying Newton’s Laws. During the pausing section, no motion occurs, thus the displacement of the rotor is a constant.

The time interval Δ*t*_2_ can be calculated by

(11) $$\Delta t_{2}=\frac{\omega (T_{1})}{\varepsilon_{d}}$$

with

(12) $$\omega (T_{1})=\frac{s_{0}}{R}\cdot \frac{n}{T_{1}}$$

The SPA will reach its maximum acceleration at time *T*_1_, which can be written as

(13) $$\varepsilon_{\max}=\frac{s_{0}}{R}\frac{n(n-1)}{T_{1}^{2}}$$

In order to ensure that the step loss is eliminated and the slipping is not generated during the rising phase, the following conditions should be satisfied,

(14) $$\Delta t_{1}<\Delta t_{2}$$

(15) $$\varepsilon_{\max}<\varepsilon_{c}$$

where Δ*t*_1_ can be obtained from experiments.

Regarding the stick–slip-shoot motion mode (i.e., Equation (15) is not satisfied), the rotor’s angular displacement in the four distinct time intervals can be calculated by the following equations,

(16) $$\begin{array}{ll} \theta (0<t<T_{4})=\frac{S(t)}{R}=\frac{S_{0}}{R}\cdot {\left(\frac{T_{4}}{T_{1}}\right)}^{n}, & \left(\mathrm{sticking}\;\mathrm{section}\right)\\ \theta (T_{4}<t<T_{1})=\theta (T_{4})+\omega (T_{4})\cdot (t-T_{4})+\frac{1}{2}\cdot \varepsilon_{d}\cdot (t-T_{4}), & \left(\mathrm{slipping}\;\mathrm{section}\right)\\ \theta (T_{1}<t<T_{3})=\theta (T_{1})+\frac{\omega {\left(T_{1}\right)}^{2}}{2\varepsilon_{b}}, & \left(\mathrm{shooting}\;\mathrm{section}\right)\\ \theta (T_{3}<t<T)=\theta (T_{3}), & \left(\mathrm{pausing}\;\mathrm{section}\right) \end{array}$$

with

(17) $$T_{4}={\left[\frac{\varepsilon_{c}\cdot R\cdot T_{1}^{2}}{s_{0}\cdot n(n-1)}\right]}^{\frac{1}{n-2}}\cdot T_{1}.$$

The average velocity can be defined as below,

(18) $$\varpi =\theta (T_{3})\cdot f=\frac{\theta (T_{3})}{T}$$

where *f* is the frequency of the driving signal.

## 4. Results and Discussion

The performance of the motor driven by the exponential signal is evaluated based on the results from the simulation and experiments in this section. The step response of the rotor with different external loads is also analyzed in the simulation. The amplitude of the driving signal is 100 V and the corresponding free stroke *S*_0_ is 0.234 μm. Driving signals with different curve shapes and external loads from 0 g to 1.5 g are employed in the simulation. The radius of the rotor *R* is 2 mm.

**Figure 7:**
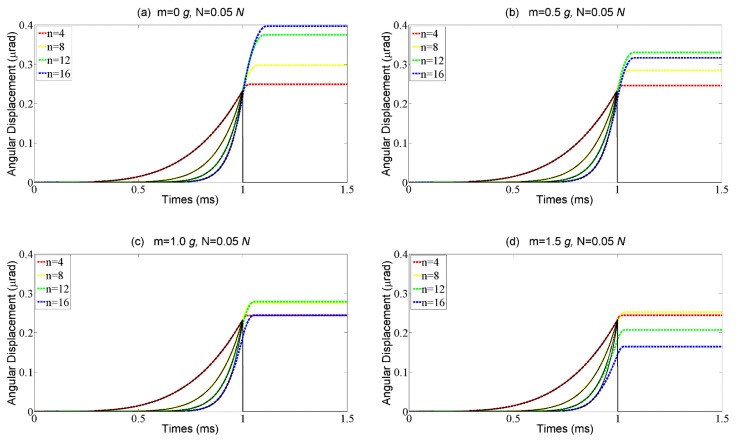
The rotor angular displacement vs different driving signals described by the base numbers with four external loads, (**a**) m = 0 g; (**b**) m = 0.5 g; (**c**) m = 1 g; (**d**) m = 1.5 g.

The results of the numerical simulation are shown in Figure 7. When the external load is 0 g, the rotor’s response follows the stick–shoot motion mode, and the overshoot is very large, as shown in Figure 7a. With the external load increasing, the step response to the abrupter curves turns to follow the stick–slip-shoot motion mode, in which the step size decreases sharply especially when the base number of the signal is large, as shown in Figure 7b. When the external load further increases, although the step loss does not exist, the step size is still smaller than the free stroke *S*_0_, as shown in Figure 7c,d. Therefore, to achieve a large step size, the stick–slip-shoot motion mode should be avoided (i.e., a larger base number of the curve is not always better).

**Figure 8:**
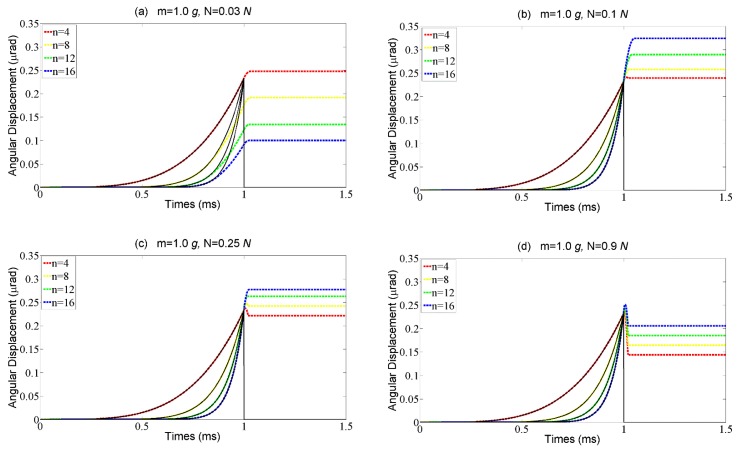
The rotor angular displacement vs different driving signals described by the base numbers with four external loads, (**a**) N = 0.03 g; (**b**) N = 0.1 g; (**c**) N = 0.25 g; (**d**) N = 0.9 g.

According to the theoretical model established above, the preload *F_pre_* is also important for the motion performance of the motor, which significantly impacts on the critical acceleration in the rising phase and the acceleration of the rotor in the time interval Δ*t*_1_. The results of the numerical simulation of the rotor’s step characteristics with different preloads are shown in Figure 8. In the case of a small preload, it is hard to prevent the rotor from slipping during the rising time even though the driving signal is a slightly abrupt curve, which goes against a large step size, as shown in Figure 8a. With the preload increasing to some extent, the rotor can stick to the SPA during the rising phase and the step size increases due to the noteworthy overshoot, as shown in Figure 8b. When the preload further increases, the distance of the overshoot decreases while the step loss appears, as shown in Figure 8c,d. It is because the preload increases not only the critical acceleration in the rising phase, but also the acceleration in the time interval Δ*t*_1_, which makes the condition in Equation (15) unsatisfied. Therefore, the same as the base number, the preload should also be carefully designed according to the external load.

**Figure 9:**
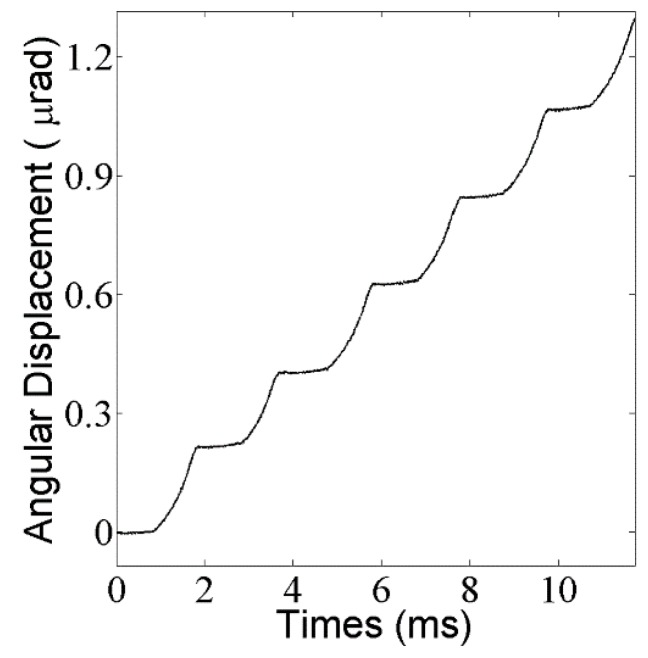
Results from the experiment.

**Figure 10:**
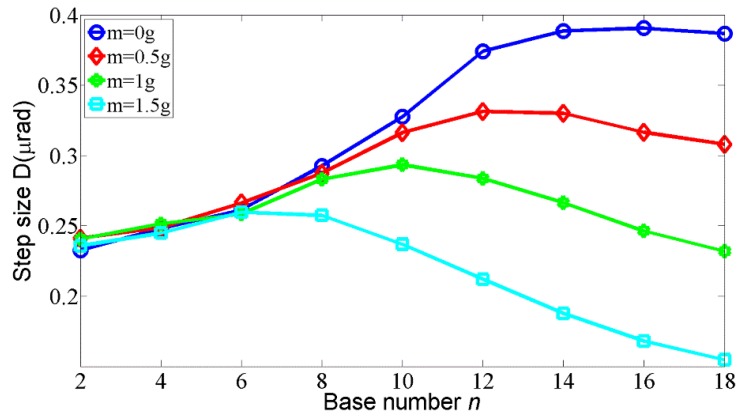
Step size D vs the base number *n.*

The aim of the experiments is to validate the numerical simulation presented above. The step response with the external load of 1 g and the base number of 10 is presented in Figure 9, where the step loss is eliminated. A more detailed relation between step size and base number with different external loads is shown in Figure 10. As expected, the step size increases with the base number increasing before the optimal *n* is reached, and after that critical point, the step size begins to decrease. With the external load increasing, the optimal n decreases. Synthetically considering the results from the experiments and the simulations, the base number from 4 to 10 and the preload from 0.04 N to 0.08 N are considered suitable in the case of 1 g of external load. Note that the maximum error in the experiment is 15%, which results from the simplification of the theoretical model, the machining and assembly error of the prototype, and the errors in input parameters, such as friction coefficients and preload force.

The average velocity of the motor can be obtained through the results in Figure 10 by multiplying the step size by the frequency of the driving signal which is 500 Hz in the experiment. The maximum average velocity is 185 μrad/s with the base number *n* = 16 in the case of no external load. According to [17], the average velocity can be further increased by reducing the pausing time, in which case a ‘slip–slip’ motion mode will appear. This will be employed in our future work to optimize the driving curve for the rotary motor.

According to the theoretical model and the results from the simulations and experiments, it can be seen that the motion performance of the rotary motor is affected by the preload and the external load, rather than the structure sizes of the motor. In other words, the motor can, to some extent, be downsized according to the actual requirement without impacting the motion performance.

## 5. Conclusions

A friction–inertial based rotary motor actuated by SPAs is proposed in this paper, which is superior in terms of its high resolution, compact size, high load capacity, and low cost. The rotary motor was designed and fabricated to systematically study the motion performance with external load applied. According to the experiments, the saw-tooth wave was unsuitable because it is unable to restrain the aggravated step loss. To address this problem, the power-function-shape signal was selected to drive the motor to obtain a large step size by eliminating the step loss. Based on the step characteristics under this driving signal, two motion modes are proposed in this paper, namely, the stick–shoot motion mode and the stick–slip-shoot motion mode. The former motion mode can realize a large step size while the latter one cannot due to the slip during the rising phase. In addition, preload and base number are the most important parameters for the motion performance of the motor. In the future, this design has potential to be used in cases where the workspace is restricted, e.g., rotary blood pumps and the rotary tip of a flexible needle; it can also be used in the case where motion accuracy is required, e.g., the precision positioning stage.

## Nomenclature

*ϕ*	The actual step size of the rotor.
*ϕ*_0_	The ideal step size during the sticking phase.
*a*	The motion caused by the inertial effect.
*b*	The step loss during the backward motion.
*ε*	The actual angle acceleration.
*ε*_c_	The critical angle acceleration.
*ε*_b_	The angle acceleration during the backward motion.
*ε*_max_	The maximum angle acceleration of the rotor.
*a*_R_	The tangential acceleration of the contact point on the rotor.
*a*_S_	The tangential acceleration of the contact point on the SPA.
Δ*t*_1_(*t*_b_)	The time interval that the SPA return to the original position.
Δ*t*_2_	The time interval of the overshoot
*T*_1_	The end of the rising time.
*T*_2_	The end of the backward time.
*T*_3_	The end of the overshoot time.
*T*_4_	The beginning of the slipping time.
*T*	The repetition period.
R	The radius of the rotor.
*I*	The inertia momentum of the rotor.
*M*(*t*)	The actual moment of force on the rotor.
*M*_load_	The moment of force caused by the load.
*M*_max_	The maximum moment of force on the rotor.
*F*_load_	The force caused by the load.
*F*_pre_	The preload acted on the rotor.
*f*_s_	The static friction coefficient.
*f*	The frequency of the driving signal.
*θ*	The angle displacement of the rotor.
ω	The angle velocity of the rotor.
